# Foreign Bodies Left in the Abdomen after Operation

**Published:** 1898-05-14

**Authors:** 


### FOREIGN BODIES LEFT IN THE ABDOMEN AFTER OPERATION.

The New York Pathological Society recently received a report of two cases in which substances left in the abdominal cavity after operation had perforated the intestines.

In one case a piece of gauze had been used to lightly pack the pelvis, after total extirpation of the uterus and adnexa for cancer of the uterus. The house surgeon had been instructed to remove the tampon per vaginam three days after the operation, but it was not noted that in doing this he had inserted a fresh piece.

Although the patient recovered perfectly from the operation she suffered from griping pain and constipation, and after about two months she passed a strip of gauze with a *fæcal* evacuation, after which the constipation and pain entirely disappeared.

The other case was one of abdominal hysterectomy for fibromyoma.

After the operation constant pain was complained of between the umbilicus and the epigastrium, where a swelling ultimately developed which appeared to consist of adherent coils of intestine.

After many months she died soon after an operation, done with the hope of relieving an abdomino-intestinal fistula which had resulted. In this operation, after an unusually difficult separation of adhesions, a portion of distended bowel presented itself, and on examination of a rent in the intestine near this place, a piece of discoloured gauze was discovered, which, on being withdrawn, was found to be a large gauze serviette, which had evidently been left in the abdominal cavity at the time of the original operation, and had made its way into the bowel.

It would seem that the danger of leaving things in the abdomen, one which has been recognised from the early days of ovariotomy, is even more difficult to guard against now. Great as is the convenience and utility of pressure forceps, there is no doubt that they are all too apt to be lost sight of unless a very careful count is made, and the use of the temporary tampon is also by no means free from the same danger unless care is taken that it is all in one piece, and that a strip is left protruding for some distance from the abdominal cavity. It was a simple affair to count sponges, but with tampons and gauze compresses error easily creeps in.

